# Wellbeing after Brief Alcohol Interventions in Male Inpatients in a General Hospital in Singapore

**DOI:** 10.1192/j.eurpsy.2024.855

**Published:** 2024-08-27

**Authors:** Z. W. Lew, C. S. Lim, H. S. Ong, R. M. Ong, Y. C. Ng, W. L. Teo, L. H. Peh

**Affiliations:** Department of Psychological Medicine, Changi General Hospital, Singapore, Singapore

## Abstract

**Introduction:**

Harmful alcohol consumption has significant cost on health and is associated with lower quality of life (e.g., Lu *et al.* BMC Public Health 2022; 22:789). In Singapore, a significant proportion of the adult population exhibit alcohol misuse behaviours (e.g., Lim *et al.* BMC Public Health 2013; 13:992). Many patients admitted into general hospitals have excessive alcohol consumption and related problems. These admissions can be an opportunity for intervention due to accessibility to the individuals and their time (Saitz *et al.* Ann Intern Med 2007; 146 167-176). Some studies have suggested that brief alcohol interventions (BAI) delivered in general hospitals can be effective in reducing alcohol use. However, there has been less support for the benefits of BAI on wellbeing.

**Objectives:**

This study investigated the effectiveness of BAI in improving perceived sense of wellbeing among male alcohol users admitted to a general hospital in Singapore.

**Methods:**

108 male inpatients in various medical wards received BAI by the hospital’s addiction counsellors and completed the Personal Wellbeing Index (PWI) questionnaire. At a one-year follow-up via telephone, the PWI was again administered.

**Results:**

Average PWI scores were higher at follow-up (*M* = 7.83, *SD* = 1.16) than during baseline admission (*M* = 7.60, *SD* = 1.12), *p* < 0.01. Further analyses found that scores improved significantly on PWI items related to standard of living (*M* = 7.36, *SD* = 1.41 vs *M* = 7.09, *SD* = 1.65; *p* < 0.05), health (*M* = 7.42, *SD* = 1.74 vs *M* = 6.62, *SD* = 1.87; *p* < 0.01) and achievement (*M* = 7.43, *SD* = 1.44 vs *M* = 6.98, *SD* = 1.64; *p* < 0.01). There were no significant differences in scores on the other PWI items between baseline and follow-up.

**Conclusions:**

Conclusions: The results suggest that BAI can be beneficial in improving patients’ sense of wellbeing.

**Disclosure of Interest:**

None Declared

